# Exploring the pathogen diagnosis and prognostic factors of severe COVID-19 using metagenomic next-generation sequencing: A retrospective study

**DOI:** 10.5937/jomb0-49102

**Published:** 2024-06-15

**Authors:** Weizhong Zeng, Yanchao Liang, Xiaoyuan He, Fangwei Chen, Jiali Xiong, Zhenhua Wen, Liang Tang, Xun Chen, Juan Zhang

**Affiliations:** 1 Zhuzhou Central Hospital, Department of Intensive Care Unit, Zhuzhou, China; 2 Zhuzhou Central Hospital, Department of Respiratory and Critical Care Medicine, Zhuzhou, China; 3 Zhuzhou Central Hospital, Nursing Department, Zhuzhou, China; 4 Zhuzhou Central Hospital, Department of Rheumatology and Immunology, Zhuzhou, China; 5 Zhuzhou Central Hospital, Department of General Internal Medicine, Zhuzhou, China; 6 Zhuzhou Central Hospital, Department of Hepatobiliary and Pancreatic Surgery, Zhuzhou, China; 7 Zhuzhou Central Hospital, Reproductive center, Zhuzhou, China

**Keywords:** pathogens, mNGS, conventional pathogen test, APACHCE-II, SOFA, patogeni, mNGS, konvencionalni test na patogene, APACHCE-II, SOFA

## Abstract

**Background:**

This study aimed to identify pathogens and factors that predict the outcome of severe COVID-19 by utilizing metagenomic next-generation sequencing (mNGS) technology.

**Methods:**

We retrospectively analyzed data from 56 severe COVID-19 patients admitted to our hospital between December 2022 and March 2023. We analyzed the pathogen types and strains detected through mNGS and conventional microbiological testing and collected general patient information.

**Results:**

In this study, 42 pathogens were detected using mNGS and conventional microbiological testing. mNGS had a significantly higher detection rate of 90.48% compared to 71.43% for conventional testing (P=0.026). A total of 196 strains were detected using both methods, with a significantly higher detection rate of 70.92% for mNGS compared to 49.49% for conventional testing (P=0.000). The 56 patients were divided into a survival group (33 cases) and a death group (23 cases) based on clinical outcomes. The survival group had significantly lower age, number of pathogens detected by mNGS, number of pathogens detected by conventional testing, APACHE-II score, SOFA score, high-sensitivity troponin, creatine kinase-MB subtype, and lactate dehydrogenase compared to the death group (P<0.05). Multivariate logistic regression analysis showed that these factors were risk factors for mortality in severe COVID-19 patients (P<0.05). In contrast, ROC curve analysis revealed that these factors had diagnostic values for mortality, with AUC values ranging from 0.657 to 0.963. The combined diagnosis of these indicators had an AUC of 0.924.

**Conclusions:**

The use of mNGS technology can significantly enhance the detection of pathogens in severe cases of COVID-19 and also has a solid ability to predict clinical outcomes.

## Abstract (sr)

**Uvod:**

Ova studija je imala za cilj da identifikuje patogene i faktore koji predviđaju ishod teškog COVID-19 korišćenjem tehnologije metagenomskog sekvenciranja sledeće generacije (mNGS).

**Metode:**

Retrospektivno smo analizirali podatke od 56 teških pacijenata sa COVID-19 primljenim u našu bolnicu između decembra 2022. i marta 2023. Analizirali smo tipove i sojeve patogena otkrivene putem mNGS i konvencionalnog mikrobiološkog testiranja i prikupili opšte informacije o pacijentima.

**Rezultati:**

U ovoj studiji, 42 patogena su otkrivena korišćenjem mNGS i konvencionalnog mikrobiološkog testiranja. mNGS je imao značajno veću stopu detekcije od 90,48% u poređenju sa 71,43% za konvencionalno testiranje (P=0,026). Ukupno 196 sojeva je otkriveno korišćenjem obe metode, sa značajno većom stopom detekcije od 70,92% za mNGS u poređenju sa 49,49% za konvencionalno testiranje (P=0,000). 56 pacijenata je podeljeno u grupu za preživljavanje (33 slučaja) i grupu sa smrću (23 slučaja) na osnovu kliničkih ishoda. Grupa za preživljavanje imala je značajno nižu starost, broj patogena otkrivenih pomoću mNGS-a, broj patogena otkrivenih konvencionalnim testiranjem, APACHE-II skor, SOFA skor, troponin visoke osetljivosti, podtip kreatin kinaze-MB i laktat dehidrogenazu u poređenju sa grupom smrti. (P<0,05). Multivarijantna logistička regresiona analiza je pokazala da su ovi faktori faktori rizika za smrtnost kod teških pacijenata sa COVID-19 (P<0,05). Nasuprot tome, analiza ROC krive je otkrila da ovi faktori imaju dijagnostičke vrednosti mortaliteta, sa vrednostima AUC u rasponu od 0,657 do 0,963. Kombinovana dijagnoza ovih indikatora imala je AUC od 0,924.

**Zaključak:**

Upotreba mNGS tehnologije može značajno poboljšati otkrivanje patogena u teškim slučajevima COVID-19 i takođe ima solidnu sposobnost predviđanja kliničkih ishoda.

## Introduction

COVID-19 is a pneumonia caused by the highly contagious novel coronavirus SARS-CoV-2, leading to an unprecedented global public health crisis. This pandemic has placed immense pressure on health systems worldwide and has severely impacted the global economy. In severe pneumonia cases, mechanical ventilation may be necessary due to severe bilateral lung infection, cardiopulmonary failure, and bronchial constriction. COVID-19-related pneumonia is particularly concerning as it has a higher mortality rate and poorer prognosis. It is also prone to recurrent upper respiratory tract infections, which can limit exercise tolerance in surviving patients [1]. Clinical studies have established a clear correlation between age and multi-organ dysfunction as negative prognostic factors in severe COVID-19 pneumonia. However, further research is required to develop effective methods for diagnosing, treating, and assessing the prognosis of pathogen infections associated with this disease [2] [3]. Historically, the detection of pathogens in medical research relied upon culture, immunohistochemistry, and molecular biology techniques. Although these methods have been valuable, they present significant challenges, such as the need for specialized specimen preparation and extraction and limitations in providing accurate diagnoses and distinguishing specific pathogen infections [4] [5]. Metagenomic next-generation sequencing (mNGS) is a pathogen detection method that does not rely on culture and can directly extract microbial DNA from environmental or clinical specimens. It utilizes metagenomic sequencing to detect and study microbial genetic composition and community function [6]. This study retrospectively investigates the factors influencing the diagnosis and prognosis of severe COVID-19 pneumonia using mNGS technology, aiming to improve the prognosis of this disease.

## Materials and methods

### Clinical data

A retrospective analysis was conducted on the data of 56 patients with severe COVID-19 pneumonia admitted to our hospital from December 2022 to March 2023. Among them were 43 males and 13 females, with an age range of 38 to 75 years and an average age of (56.49±8.45) years. (1) Inclusion criteria: Patients or their family members have signed informed consent forms and meet the diagnostic criteria for severe COVID-19 pneumonia [7], which included meeting any of the first two criteria and any of the last seven criteria in the following: 1) Need for tracheal intubation and mechanical ventilation; 2) After fluid resuscitation for septic shock, still requiring vasopressor therapy; 3) Respiratory rate ≥ 30 breaths/min; 4) Oxygenation index 250 mmHg; 5) Impaired consciousness or orientation; 6) Systolic blood pressure < 90 mmHg, central body temperature < 36 , requiring aggressive fluid resuscitation; 7) Blood urea nitrogen ≥ 20 mg/dL; 8) WBC > 10×10^9^/L or < 4×10^9^/L; 9) Multilobar infiltrates or interstitial changes in lung tissue visible on lung CT, with patchy, plaque-like infiltrating shadows appear, which may be accompanied by pleural effusion. (2) Exclusion criteria: 1) Presence of non-infectious interstitial lung disease or malignant lung tumor; 2) History of thoracic or pulmonary surgery, severe mental illness, immune system disorders, congenital and genetic disorders; 3) Recurrent infection after treatment for severe pneumonia or infectious diseases; 4) Death within 24 hours of admission, transfer to another hospital midway, or discharge at the request of family members; 5) Patients transferred from another hospital with incomplete clinical data.

### Conventional pathogen test

(1) Secretion culture: Deep sputum was coughed out forcefully after rinsing the mouth with physiological saline twice in the morning. Approximately 1~2 mL of sputum was then collected using a sterile sampling cup and immediately sent for sputum culture, smear, and acid-fast staining. (2) Serological test: 10 mL of venous blood was collected from the patient while fasting, centrifuge at 3000 r/min for 10 min, take the upper serum, and perform aspergillus galactomannan (GM) detection, 1,3-β-D glucan (G) detection, detection of DNA sequences of mycobacterium tuberculosis, respiratory pathogen IgM antibody detection (mainly including Legionella pneumophila, respiratory syncytial virus, adenovirus, Mycoplasma pneumoniae, Chlamydia pneumoniae, influenza A virus, influenza B virus, Coxsackie virus and parainfluenza virus types 1, 2 and 3).

### mNGS

Under an aseptic procedure, we insert the entry of the bronchus or the entrance of the subsegment of the affected lung lobe. Inject 37°C saline through the bronchoscope and then aspirate two tubes of bronchoalveolar lavage fluid (BALF) of 5 mL each for rapid low-temperature preservation (4°C). One tube was sent to our hospital’s laboratory for secretion culture; the other was transported to Tianjin Union Bojing Medical Diagnosis Technology Co., Ltd. (Tianjin, China) and Berry Genomics Co., Ltd. for metagenomic detection. The main procedures of mNGS include (1) specimen reception and information entry; (2) DNA extraction: The specimen is centrifuged at 3000 r/min for 30 min, and 0.3~0.5 mL of the sediment at the bottom is transferred to a 1.5 mL microcentrifuge tube. DNA is extracted using a genomic DNA kit for micro samples; (3) DNA processing and library construction: After DNA is sheared by ultrasound, end repair, adapter addition, and polymerase chain reaction (PCR) amplification are performed to form single-stranded DNA circles to construct a library; (4) Sequencing and information analysis: After quality control by combining PCR quantification and gene information annotation, DNA sequencing is performed with single-end read. We also filtered sequences with length <35 bp, low quality, complexity, and repeat sequences. We removed human host sequences hg19 and YH. Compare the remaining sequences with the reference database to query sequences with high similarity and coverage to confirm all possible pathogens that cause severe pneumonia. Use gene sequencing software to calculate the sequencing depth and coverage of the pathogenic microorganism, then include RNA viruses in the analysis.

### Observation indicators

Compared the types and strains of pathogens detected by the two methods and collected general information about patients, including sex, age, hospitalization time, mechanical ventilation time, Acute Physiology and Chronic Health Evaluation-II (APACHCE-II), Sequential Organ Failure Assessment (SOFA), etc. (1) APACHCE-II: Includes rectal temperature, respiration rate, heart rate, mean arterial pressure, oxygenation index, blood creatinine level, serum sodium level, serum potassium level, pH value, white blood cell count (WBC), hematocrit (Hct), bicarbonate (HCO3-)12 physiological variables scoring with a score of 0–60 points; age score of 0–6 points; chronic health status (CPS) score of 2–5 points; total score of 0–71 points; the higher the score, the higher the mortality rate. (2) SOFA: Includes respiration rate, coagulation system function level, liver function level, circulatory system function level, nervous system function level, and kidney function level with six items, each scoring four points; total score of 0–24 points; the higher the score, the worse the prognosis.

### Statistical analysis

We employed Statistic Package for Social Science (SPSS)27.0 software (IBM, Armonk, NY, USA) for data process and rectification; entered count data in »n(%)« format; chi-square test for two independent sample rates/composition ratios was performed; entered measurement data in »x̄±s« format; paired t-test for group comparison Use paired t-test for within-group comparison were used; multi-factor Logistic regression analysis was used to analyze the factors affecting death in patients with severe pneumonia; we also utilized the receiver operating characteristic curve (ROC) area to analyze the predictive value of various indicators; P<0.05 indicates statistical significance.

## Results

### Comparison of pathogen species detection between two methods

A total of 42 pathogens were detected by the two methods, including 25 bacteria, six fungi, eight viruses, and three prokaryotes. The detection rate of mNGS (90.48%) was significantly higher than that of the conventional pathogen test (71.43%), and the difference was statistically significant (*P*<0.05), as shown in Table 1.

**Table 1:** Comparison of Pathogen Detection between the Two Methods [cases/ (n %)].

Group	Bacteria	Fungi	Viruses	Prokaryotes	Total
mNGS	22(52.38)	5(11.90)	8(19.05)	3(7.14)	38(90.48)
Conventional	16(38.10)	5(11.90)	7(16.67)	2(4.76)	30(71.43)
χ^2^	1.730	0.000	0.081	0.213	4.941
P	0.188	1.000	0.776	0.645	0.026

### Comparison of the number of pathogen strains detected by two methods.

**Figure 1:**
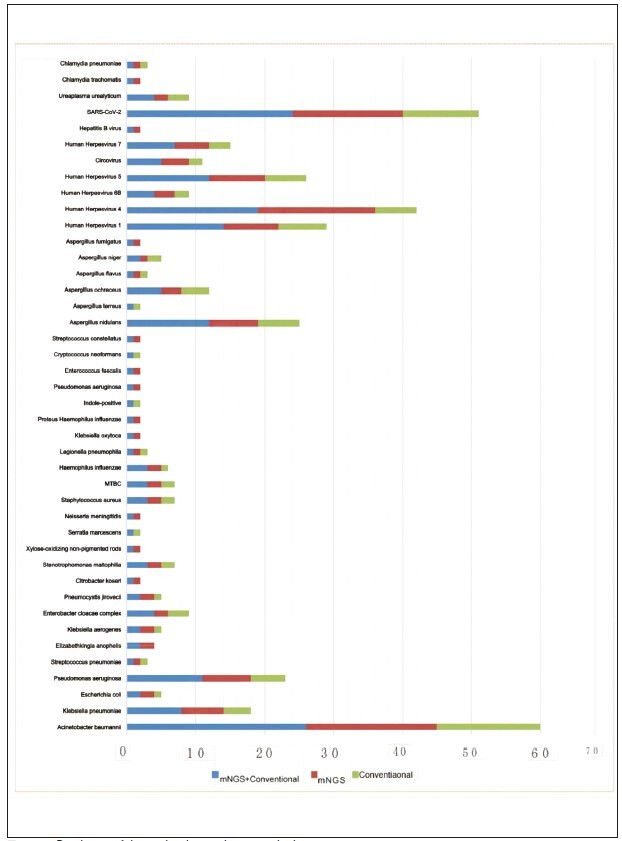
Distribution of detected pathogens by two methods.

The two methods detected a total of 196 pathogen strains. The mNGS method detected 139 strains, with a detection rate of 70.92%. The conventional pathogen test method detected 97 strains, with a detection rate of 49.49%, significantly lower than mNGS, and the difference was statistically significant (χ^2^=18.782, *P*<0.001). The highest detection rate by mNGS was for Acinetobacter baumannii at 9.69% (19/196), followed by Human Herpesvirus 4 at 8.67% (17/196) and SARS-CoV-2 at 8.16% (16/196). In contrast, the highest detection rate by the conventional pathogen detection method was for Acinetobacter baumannii at 7.65% (15/196), followed by SARS-CoV-2 at 5.61% (11/196) and Human Herpesvirus 1 at 3.57% (7/196). The specific distribution of pathogens can be seen in Figure 1.

### Grouping and general information comparison

The patients were divided into two groups based on their final clinical outcomes: a survival group of 33 cases and a death group of 23. There were no statistical differences (P>0.05) in terms of gender, length of hospital stay, duration of mechanical ventilation, pathogenic species detected by mNGS, pathogenic species detected by conventional pathogen testing, creatine kinase, C-reactive protein, IL-6, IL-17, and TNF-α between the two groups. However, the survival group had significantly lower age, number of pathogens detected by mNGS, number of pathogens detected by conventional pathogen testing, APACHCE-II, SOFA, high-sensitivity troponin, creatine kinase-MB subtype, and lactate dehydrogenase compared to the death group, with statistically significant differences (*P*<0.05), as shown in Table 2.

**Table 2:** Comparison of general data between the two groups [case/ (n %), ±s].

Item		Survival Group (n=33)	Death Group (n=23)	χ^2^/t	P
Sex	Male	26	17	0.181	0.671
	Female	7	6		
Age		52.43±7.54	57.26±8.29	2.264	0.028
Length of stay (d)		8.56±2.45	8.39±2.62	0.248	0.805
Duration of mechanical ventilation (d)		5.38±1.87	5.46±2.05	0.151	0.880
Types of pathogenic species (mNGS)		2.58±1.03	2.34±1.08	0.841	0.404
Number of pathogenic species (mNGS)		1.57±0.41	3.17±0.82	9.638	<0.001
Types of pathogenic species (conventional)		1.53±0.62	1.79±0.70	1.464	0.149
Number of pathogenic species (conventional)		0.87±0.34	1.15±0.46	2.621	0.011
APACHCE-II scores		22.65±7.49	29.75±6.24	3.730	0.001
SOFA scores		8.97±1.68	10.32±2.14	2.642	0.011
High-sensitivity troponin (pg/mL)		0.09±0.02	0.18±0.08	6.213	<0.001
Creatine kinase (U/L)		46.53±7.22	47.28±8.25	0.361	0.720
Creatine kinase-MB subtype (U/L)		10.19±1.18	16.05±4.45	7.234	<0.001
Lactate dehydrogenase (U/L)		294.35±46.21	408.73±82.15	6.646	<0.001
Lactate dehydrogenase (U/L)		58.70±10.25	60.15±11.03	0.505	0.616
IL-6 (pg/mL)		28.39±5.48	29.17±6.20	0.497	0.622
IL-17 (pg/mL)		18.56±3.47	19.48±4.13	0.903	0.371
TNF-α (pg/mL)		1.57±0.43	1.62±0.45	0.420	0.676

### Multivariable logistic regression analysis for risk factors of mortality in patients with severe pneumonia

Table 3 and Table 4 reveal that age, the number of pathogenic species detected by mNGS, the number of pathogenic species detected by conventional pathogen testing, APACHE-II score, SOFA score, high-sensitivity troponin, creatine kinase-MB subtype, and lactate dehydrogenase are all significant risk factors for mortality in patients with severe pneumonia (*P*<0.05).

**Table 3:** Variable Assignment.

Variable	Assignment
Age	0: <60 year; 1: ≥60 year
Number of pathogenic<br>species (mNGS)	0: < 2 strains; 1: ≥ 2 strains
Number of pathogenic<br>species (conventional)	0: < 2 strains; 1: ≥ 2 strains
APACHCE-II	0: < 25 scores; 1: ≥ 25 scores
SOFA	0: < 10 scores; 1: ≥ 10 scores
High-sensitivity troponin	0: < 0.1 pg/mL; 1: ≥ 0.1 pg/mL
Creatine kinase-MB subtype	0: < 12 U/L; ≥ 12 U/L
Lactate dehydrogenase	0: < 300 U/L: ≥ 300 U/L

**Table 4:** Multivariable logistic regression analysis for mortality in patients with severe pneumonia.

Factors	β	SE	Wald2	OR	P	95%CI
Age ≥60 year	1.064	0.386	7.141	2.897	0.006	1.212∼9.151
Number of pathogenic species (mNGS) ≥ 2<br>strains	1.392	0.317	13.852	4.023	<0.001	2.176∼12.760
Pathogenic species (conventional) ≥ 2 strains	0.945	0.458	4.505	2.572	0.037	0.917∼6.721
APACHCE-II score≥25	1.143	0.345	9.603	3.135	<0.001	1.957∼11.036
SOFA score≥10	1.178	0.329	10.883	3.249	<0.001	1.712∼9.525
High-sensitivity troponin ≥0.1 pg/mL	1.355	0.435	7.161	3.875	0.004	1.357∼9.876
Creatine kinase-MB subtype ≥ 12 U/L	1.376	0.417	7.913	3.958	0.001	1.436∼10.104
Lactate dehydrogenase ≥ 500 U/L	1.165	0.428	6.360	3.206	0.011	1.002∼7.839

### Value analysis of various indicators in predicting mortality in patients with severe pneumonia

**Figure 2:**
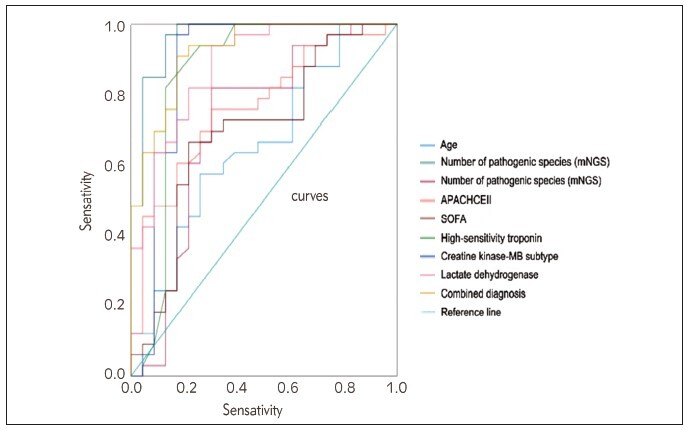
ROC Curve analysis of various indicators for predicting mortality in severe pneumonia patients.

The analysis of the ROC curve reveals that certain factors can help predict the mortality of severe pneumonia patients. These factors include age, the number of pathogenic species detected by mNGS and conventional pathogen testing, APACHE-II score, SOFA score, high-sensitivity troponin, creatine kinase-MB subtype, and lactate dehydrogenase. According to the results, the AUC values for each indicator vary, ranging from 0.657 to 0.963. However, when combined, these indicators have an impressive AUC of 0.924. These findings are presented in Table 5 and Figure 2.

**Table 5:** Sensitivity, specificity, and AUC for each indicator.

Indicator	Sensitivity (%)	Specificity (%)	AUC	95%CI
Age	57.60	73.90	0.657	0.535∼0.778
Number of pathogenic species <br>(mNGS)	97.00	87.00	0.963	0.926∼1.000
c	81.80	69.60	0.713	0.597∼0.830
APACHCE-II	75.80	69.60	0.764	0.655∼0.872
SOFA	66.70	88.30	0.694	0.575∼0.814
High-sensitivity troponin	81.80	87.00	0.857	0.765∼0.949
Creatine kinase-MB subtype	97.00	82.60	0.883	0.799∼0.966
Lactate dehydrogenase	93.90	69.60	0.880	0.808∼0.952
Combined diagnosis	90.90	82.60	0.924	0.869∼0.978

## Discussion

Respiratory tract infections are responsible for the most fatalities among all infectious diseases. Despite the widespread use of molecular biology methods, a significant number of respiratory tract infections remain unidentified for the associated pathogens due to the diverse range of relevant pathogens [8] [9]. Conventional detection methods typically take several years to complete human genome sequencing, whereas mNGS can be completed in about a week. This not only dramatically improves the speed and accuracy of sequencing but also provides necessary technical support for identifying difficult, emerging, or newly discovered pathogens [10] [11]. In this study, it was suggested that out of 56 patients, 42 pathogens were identified. The most detected pathogen through both methods was Acinetobacter baumannii, which is known for its resistance to multiple drugs and ability to strong survival ability [12]. The pathogen detection rate of mNGS is significantly higher than that of conventional pathogen detection methods, indicating a greater advantage in diagnosing severe COVID-19 pneumonia. Chen Tingting et al. [13] found that compared to culture and serological tests, mNGS has a significantly higher detection rate for bacteria and can simultaneously sequence millions of DNA molecules without being affected using hormone medications by the host. Severe pneumonia patients often have multiple pathogens co-infections, but a single detection method often misses or misidentifies some rare or biologically less obvious bacteria or viruses, such as Elizabethkingia anophelis, Citrobacter koseri, Huprescue virus, and Pseudomonas luteola [14] [15].

Both methods detected a total of 196 strains of pathogens, mNGS detected 139 strains, resulting in a detection rate of 70.92%., significantly higher than the conventional pathogen detection rate of 49.49%. However, 29.08% of the strains were still not detected. This analysis is related to the following factors: (1) In clinical diagnosis and treatment, the positive or negative results of mNGS in a specific site cannot be the sole basis for clinical diagnosis, which affects the diagnosis and treatment of pathogenic microorganisms [16] [17]; (2) The process of extracting, transporting, and testing BALF takes a significant amount of time and covers a large area. Incorrect preservation or handling increases the risk of decreased sensitivity and specificity. [18]; (3) Some uncommon pathogens have only 1-3 strains, and the small sample size reduces the detection rate [19]; (4) There is no unified standard for the sequencing process of mNGS, and different testing units use different reagent standards, and even DNA and RNA sequencing strategies may vary, which further affects the accuracy of the results [20] [21]; (5). The nucleic acid content of the specimen source can affect the relative abundance of pathogenic microorganisms detected by mNGS, reducing the sequencing sensitivity [22]. In response to this, Peng et al. [23] suggested combining conventional microbial detection methods to improve the diagnostic efficiency of mNGS. Secondary sequencing data analysis can also be performed to obtain more detailed genetic profiles, thereby determining drug-resistant genes of bacteria or viruses and predicting drug resistance [24]. At the same time, indicators such as APACHE-II, SOFA, and high-sensitivity troponin have considerable diagnostic value for the prognosis of severe COVID-19 pneumonia. When using mNGS technology to analyze respiratory specimens of patients, it is also necessary to complete serological tests as soon as possible and comprehensively evaluate the patient’s physiological indicators to assist in analyzing pathogenic microorganisms and optimize treatment strategies.

This study also has certain limitations, such as a small sample size and limited scope, which may restrict the generalizability and applicability of the research findings. Because of the retrospective design used in this study, there is also a risk of potential information bias and selection bias. Future research needs to overcome these limitations by using a larger sample size, involving multiple centers, adopting a prospective design, and conducting more in-depth analysis of the relationship between pathogens and diseases to further validate and deepen the findings of this study.

It is important to note that mNGS technology excels in identifying a broader range of harmful organisms compared to conventional pathogen detection methods. Healthcare providers must comprehensively understand COVID-19 pneumonia’s infectious characteristics and prevalence to achieve a prompt and precise detection rate. However, there is no definitive consensus on the timing of specimen collection and how to eliminate interference from high background human nucleic acids, further advancements in sequencing technology must be conducted to enhance the accessibility of this technology in clinical field.

## Dodatak

### Conflict of interest statement

All the authors declare that they have no conflict of interest in this work.
